# Takayasu Arteritis–Induced Acute Myocardial Infarction: Coronary Imaging- and Physiology-Guided Management

**DOI:** 10.1016/j.jaccas.2025.106586

**Published:** 2026-01-21

**Authors:** Dharmaraj Karthikesan, Yuen Hoong Phang, Kenneth Kay Leong Khoo, Chee Tat Liew, Saravanan Krishinan, Kantha Rao Narasamuloo, Vijayendran Rajalingam, Kai Soon Liew, Bharat Khialani, William Kongto Hau

**Affiliations:** a Department of Cardiology, Hospital Sultanah Bahiyah, Alor Setar, Kedah, Malaysia; b Pantai Hospital Penang, Penang, Malaysia; c Tan Tock Seng Hospital, National Healthcare Group, Singapore; d The Chinese University of Hong Kong, Hong Kong SAR, China

**Keywords:** case report, intravascular ultrasound, myocardial infarction, Takayasu arteritis, vasculitis

## Abstract

**Background:**

Myocardial infarction (MI) in young women may result from vasospasm, spontaneous coronary artery dissection, vasculitis, or plaque rupture. These etiologies are not always distinguishable on coronary angiography alone, necessitating additional intravascular imaging.

**Case Summary:**

A 25-year-old woman presented with non–ST segment elevation MI and was found to have proximal left anterior descending artery occlusion. Intravascular ultrasound (IVUS) revealed features suggestive of Takayasu arteritis, which was confirmed through further work-up. Based on IVUS and fractional flow reserve (FFR) findings, stenting was deferred. She was started on immunosuppressive therapy and remained asymptomatic at 2-month follow-up.

**Discussion:**

Multimodality assessment using IVUS together with physiologic evaluation by FFR was critical in identifying vasculitic changes and guiding a conservative interventional strategy. Long-term management focused on immunosuppression to control disease activity.

**Take-Home Messages:**

In young women with MI, nonatherosclerotic causes should be considered. IVUS and FFR can aid diagnosis and help avoid unnecessary coronary intervention.

## Visual Summary

**Figure:**
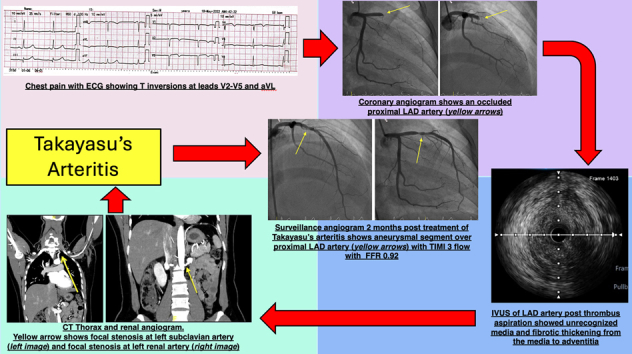

**Visual Summary:**
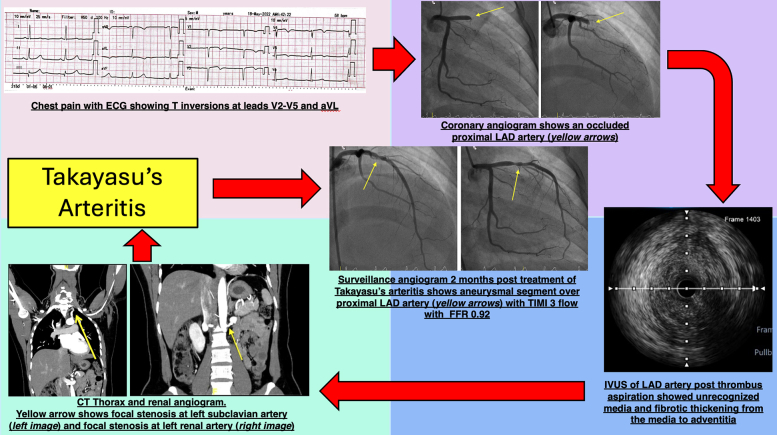
Takayasu Arteritis–Induced Acute Myocardial Infarction Successfully Managed With the Aid of IVUS and FFR CT = computed tomography; ECG = electrocardiogram; FFR = fractional flow reserve; IVUS = intravascular ultrasound; LAD = left anterior descending artery.

## History of Presentation

A 25-year-old Malay woman presented with intermittent central chest pain that worsened with exertion over the 2 days preceding admission. She denied joint pain, rash, or other systemic symptoms. Physical examination was unremarkable.

**Take-Home Messages**

• In young women with MI, the use of IVUS should be considered to rule out nonatherosclerotic causes.
• The use of IVUS and FFR can aid diagnosis of atypical coronary presentations and help avoid unnecessary coronary intervention.

## Past Medical History

At age 18 years, the patient experienced a spontaneous subdural hemorrhage. Magnetic resonance imaging of the brain at that time demonstrated features suggestive of cerebral vasculitis. However, she was lost to follow-up, and no definitive diagnosis was established.

## Differential Diagnosis

Given her presenting symptoms, an initial diagnosis of acute coronary syndrome (ACS) was considered. However, in view of her young age and prior history of possible vasculitis, coronary vasculitis was also entertained as a potential etiology.

## Investigations

**Figure 1:**
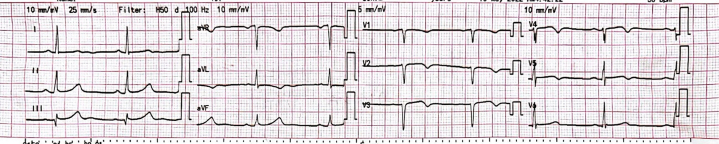
Electrocardiogram Showing T-Wave Inversions in Leads V2 to V5, and AVL

The initial electrocardiogram showed T-wave inversions in leads V_2_ to V_5_ and aVL (Figure 1). Serial troponin I levels increased from 38 to 19,000 ng/L (reference range: 0-15 ng/L). Transthoracic echocardiography demonstrated regional wall motion abnormalities involving the apical and anteroseptal segments, with a left ventricular ejection fraction of 47% (Video 1). The patient's lipid profile and blood glucose levels were normal.

Given her presentation and the elevated troponin levels, a diagnosis of non–ST-segment elevation myocardial infarction was made.

## Management

**Figure 2:**
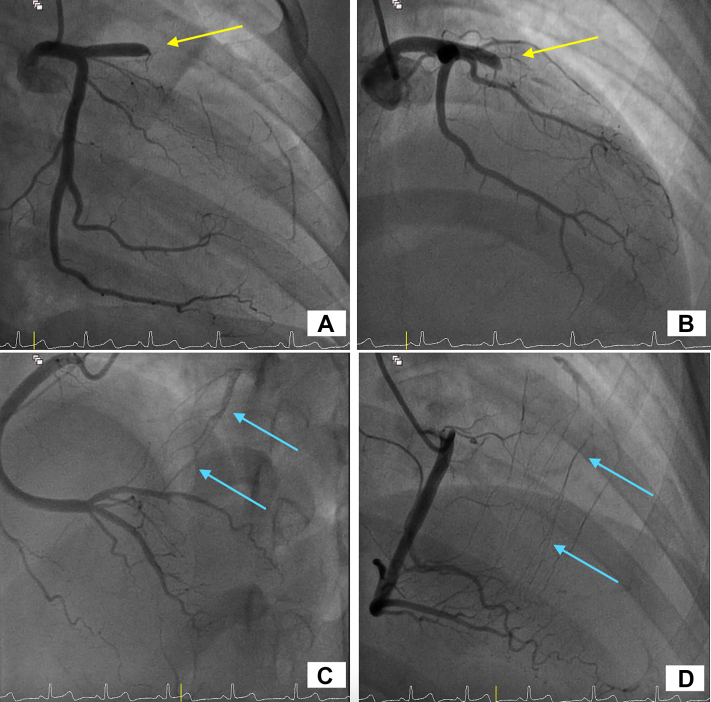
Coronary Angiography of the Left and Right Coronary Systems Coronary angiography of the left coronary system acquired at (A) right anterior oblique caudal view and (B) right anterior oblique cranial view showing proximal LAD occlusion (yellow arrows). Coronary angiography of the right coronary system acquired at (C) anteroposterior cranial view and (D) right anterior oblique view showing collaterals from posterior descending artery (blue arrows). See also Videos 2 and 3. LAD = left anterior descending artery.

**Figure 3:**
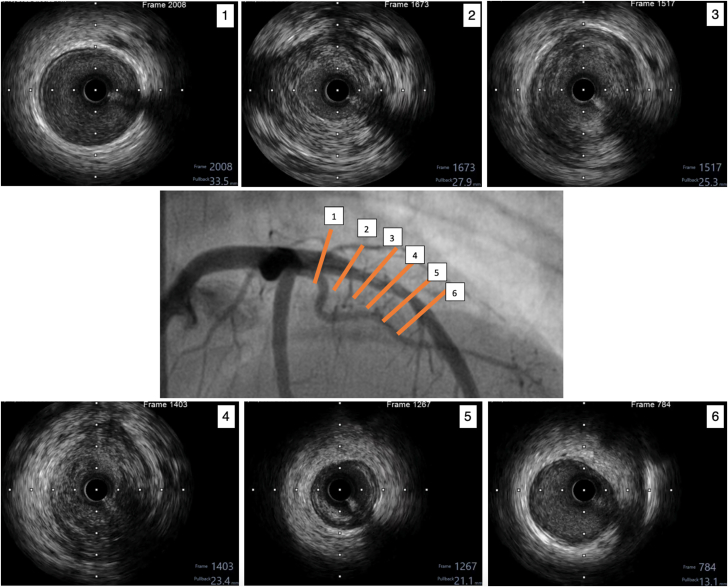
IVUS of the Proximal Left Anterior Descending Artery The center image shows the location of cuts (1) to (6). (1) Proximal segment appeared normal. (2) Area of stenosis with negative remodeling. (3 and 4) Aneurysmal segments with unrecognizable media and fibrotic thickening from the media to the adventitia. (5) Area of negative remodeling (6): Distal segment appeared normal. See also Video 5. IVUS = intravascular ultrasound.

**Figure 4:**
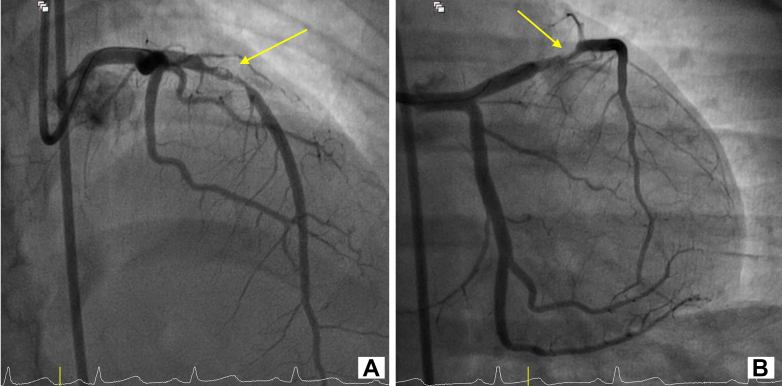
Coronary Angiography of the Left Coronary System After PCI Coronary angiography after PCI to the proximal LAD (arrows). (A) Anteroposterior cranial view and (B) right anterior oblique caudal view showing restoration of blood flow. See also Video 6. LAD = left anterior descending artery; PCI = percutaneous coronary intervention.

Urgent coronary angiography revealed an occlusion in the proximal left anterior descending artery, with collateral filling from the posterior descending artery (Figure 2, Videos 2 and 3). Because of the patient's young age and prior history suggestive of vasculitis, intravascular ultrasound (IVUS) was performed after balloon angioplasty. IVUS revealed an aneurysmal segment with intraluminal thrombus (Video 4). Thrombus aspiration was carried out, and repeat IVUS demonstrated mixed positive and negative remodeling, aneurysmal dilation, loss of media definition, and fibrotic thickening extending from the media to the adventitia (Figure 3, Video 5). Restoration of TIMI flow grade 3 was achieved after percutaneous coronary intervention (PCI) (Figure 4, Video 6).

Based on the current and past information, vasculitis was deemed the most likely etiology of her myocardial infarction (MI). As she remained pain free and stable with restored TIMI flow grade 3, stenting was deferred.

## Outcome and Follow-Up

**Figure 5:**
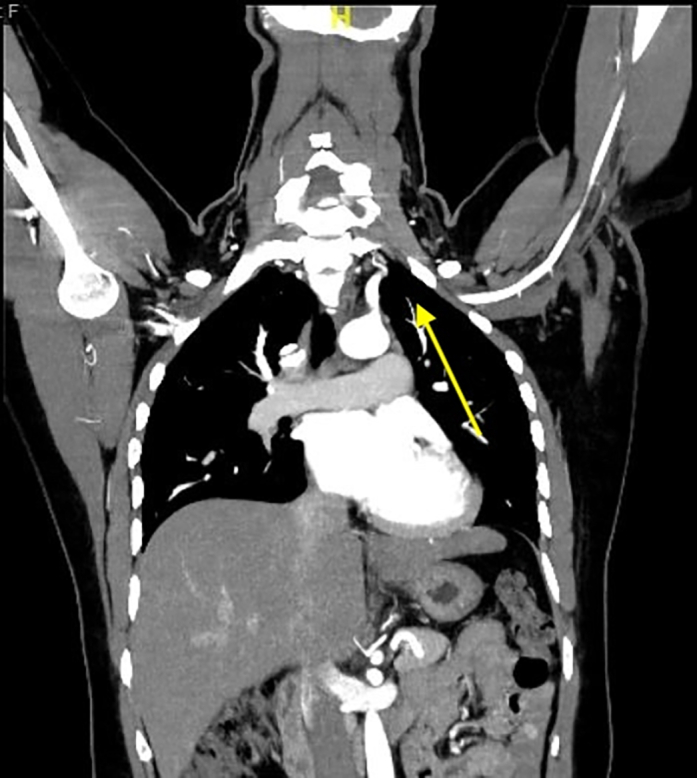
Computed Tomography of the Thorax Showing Focal Stenosis at the Left Subclavian Artery (Arrow) See also Video 7.

**Figure 6:**
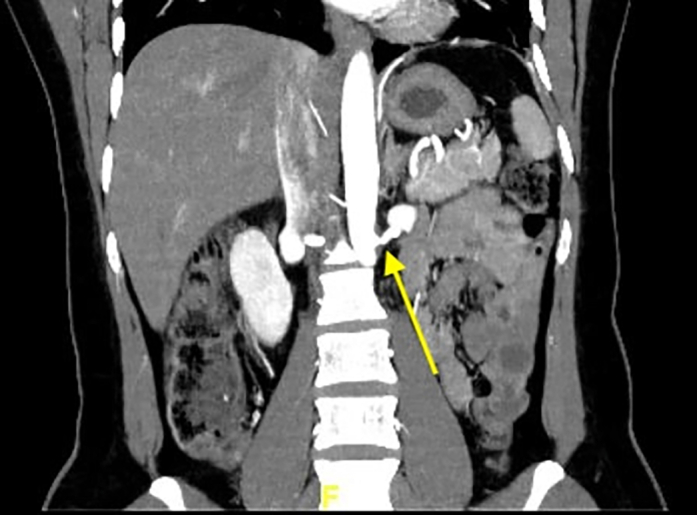
Computed Tomography Renal Angiography Showing Focal Stenosis at the Left Renal Artery (Arrow) See also Video 7.

Subsequent thoracic computed tomography and renal angiography revealed focal stenoses at the origins of the left subclavian (Figure 5, Video 7) and renal arteries (Figure 6, Video 7). Based on these findings, together with the coronary involvement and prior history, the rheumatology team established a diagnosis of Takayasu arteritis (TA) in accordance with the American College of Rheumatology criteria.^1^ The patient was started on immunosuppressive therapy in addition to aspirin, clopidogrel, and atorvastatin.

**Figure 7:**
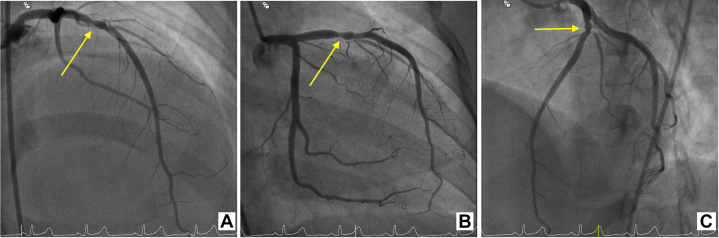
Repeat Coronary Angiography of the Left Coronary System at 2-Month Follow-Up (A) Anteroposterior cranial view, (B) right anterior oblique caudal view, and (C) left anterior oblique cranial view showing aneurysmal segment over the proximal LAD with TIMI flow grade 3. See also Video 8. LAD = left anterior descending artery.

At the 2-month follow-up, she remained asymptomatic and at NYHA functional class I status. Repeat coronary angiography demonstrated a persistent aneurysmal segment in the proximal left anterior descending artery with maintained TIMI flow grade 3 (Figure 7, Video 8). Fractional flow reserve (FFR) measured 0.92, indicating no hemodynamically significant stenosis. Therefore, PCI was deferred.

## Discussion

Advances in coronary imaging have improved detection of coronary artery disease, especially in younger patients. While traditional risk factors account for most MI, younger women may develop MI from vasculitis, spontaneous coronary artery dissection, or microvascular dysfunction. TA is a rare systemic inflammatory disease affecting large and medium-sized arteries. Coronary involvement in TA is rare, with an estimated incidence of 1 to 3 per million.^2^

TA primarily affects women younger than 40, with involvement of the thoracic aorta more common in females and abdominal aortic disease more common in males.^3^ Coronary involvement may occur via intimal hyperplasia, granulomatous arteritis, or accelerated atherosclerosis. Granulomatous arteritis typically progresses through 2 phases. In the acute phase, lymphocytic infiltration and fibroblast proliferation thicken the intima. In the chronic phase, inflammation transitions to fibrosis, replacing elastic tissue with collagen and thickening all 3 vessel layers. This may lead to aneurysm or stenosis due to mural stress.^4^

These reported pathological processes were demonstrated on our patient's IVUS findings as fibrotic intimal thickening without lipid-rich plaque, unrecognizable media layer, and transmural fibrosis extending from the intima to the adventitia, consistent with chronic vascular inflammation. IVUS can detect such changes even when angiographic findings appear normal. FFR further clarified the lesion's functional significance, supporting the decision to defer stenting. Combining IVUS and FFR offers a comprehensive assessment and may help avoid unnecessary interventions.

Formal guidelines for revascularization in TA-related coronary disease are lacking. Limited data suggest higher rates of target lesion failure and in-stent restenosis with PCI compared with coronary artery bypass grafting (CABG).^5^ However, CABG also has its own challenges, as graft vessels may be affected by vasculitis. Some studies recommend delaying surgery until inflammation is controlled. However, one study reported better outcomes with CABG versus PCI during active disease over a median follow-up of 41 months, while outcomes were similar in patients with stable TA.^6^ In addition, a 2020 case report described a successful PCI using a drug-coated balloon, with favorable outcomes at 3-year follow-up in a patient with TA-induced ACS.^7^ Although this strategy appears appealing, further studies are needed before it can be adopted widely in clinical practice.

The use of antiplatelet and antithrombotic therapy in patients with TA-related ACS has been described in case reports; however, there are no robust, disease-specific guideline recommendations to date. In the absence of such guidance, we felt it was reasonable to initiate dual antiplatelet therapy and statins in accordance with established ACS protocols. Although additional anticoagulation can be considered in selected patients, particularly those with coronary artery aneurysms or ectasia, where thrombus formation may contribute to the presentation, we elected not to pursue this strategy because the patient only had a small, isolated, single-vessel aneurysm. This approach was consistent with the medical management considerations proposed by Kawsara et al.^8^

## Conclusions

MI in young women may result from TA. In such cases, intravascular imaging modalities such as IVUS can aid in distinguishing vasculitic lesions from atherosclerotic disease. Given the increased risk of target lesion failure after PCI with stenting in Takayasu-related coronary involvement, the use of coronary imaging and physiological assessment in atypical coronary presentations is essential for guiding intervention and potentially avoiding unnecessary stenting. Furthermore, long-term control of the underlying vasculitis with appropriate immunosuppressive therapy is critical to the success and durability of any coronary intervention in this population.

## Funding Support and Author Disclosures

The authors have reported that they have no relationships relevant to the contents of this paper to disclose.

## Appendix

Video 1 Transthoracic Echocardiography Showing Regional Wall Motion Abnormalities Involving the Apical and Anteroseptal Segments, With an LVEF of 47% Parasternal long-axis, parasternal short-axis, apical 4-chamber, and apical 2-chamber views.

Video 2 Coronary Angiography of the Left Coronary System Showing Proximal LAD Occlusion Right anterior oblique caudal view and right anterior oblique cranial view.

Video 3 Coronary Angiography of the Right Coronary System Showing Collaterals From the Posterior Descending Artery Anteroposterior cranial view and right anterior oblique view.

Video 4 The First IVUS Pull-Back of the LAD Before Thrombus Aspiration Showing an Aneurysmal Segment With Intraluminal Thrombus

Video 5 The Second IVUS Pull-Back of the LAD After Thrombus Aspiration Showing Mixed Positive and Negative Remodeling, Aneurysmal Dilation, Loss of Media Definition, and Fibrotic Thickening Extending From the Media to the Adventitia

Video 6 Coronary Angiography of the Left Coronary System After PCI to the Proximal LAD Showing Restoration of Blood Flow Anteroposterior cranial view and right anterior oblique caudal view.

Video 7 CT Thorax and CT Renal Angiography Showing Focal Stenosis at Left Subclavian Artery and Left Renal Artery

Video 8 Repeat Coronary Angiography of the Left Coronary System Showing Aneurysmal Segment Over Proximal LAD With TIMI Flow Grade 3 Anteroposterior cranial view, right anterior oblique caudal view, and left anterior oblique cranial view.

## References

[1] Grayson P.C., Ponte C., Suppiah R. (2022). 2022 American College of Rheumatology/EULAR classification criteria for takayasu arteritis. Arthritis Rheumatol.

[2] Gori T. (2021). Coronary vasculitis. Biomedicines.

[3] Cobilinschi C.O., Gradinaru E., Saulescu I. (2023). Refractory takayasu’s arteritis with severe coronary involvement—case report and literature review. J Clin Med.

[4] Mohan S., Poff S., Torok K.S. (2013). Coronary artery involvement in pediatric takayasu's arteritis: case report and literature review. Pediatr Rheumatol.

[5] Jung J.H., Lee Y.H., Song G.G., Jeong H.S., Kim J.H., Choi S.J. (2018). Endovascular versus open surgical intervention in patients with takayasu's arteritis: a meta-analysis. Eur J Vasc Endovasc Surg.

[6] Wang X., Dang A., Lv N. (2017). Long-term outcomes of coronary artery bypass grafting versus percutaneous coronary intervention for takayasu arteritis patients with coronary artery involvement. Semin Arthritis Rheum.

[7] Chiew K.L.X., Lim P.O. (2021). Three-year outcome with drug-coated balloon percutaneous coronary intervention in coronary Takayasu arteritis: a case review. Catheter Cardiovasc Interv.

[8] Kawsara A., Núñez G.I., Alqahtani F. (2018). Management of coronary artery aneurysms. J Am Coll Cardiol Intv.

